# New Operational Matrices for Solving Fractional Differential Equations on the Half-Line

**DOI:** 10.1371/journal.pone.0126620

**Published:** 2015-05-21

**Authors:** Ali H. Bhrawy, Taha M. Taha, Ebrahim O. Alzahrani, Dumitru Baleanu, Abdulrahim A. Alzahrani

**Affiliations:** 1 Department of Mathematics, Faculty of Science, King Abdulaziz University, Jeddah, Saudi Arabia; 2 Department of Mathematics, Faculty of Science, Beni-Suef University, Beni-Suef, Egypt; 3 Department of Mathematics and Computer Sciences, Cankaya University, Ankara, Turkey; 4 Institute of Space Sciences, Magurele-Bucharest, Romania; Queensland University of Technology, AUSTRALIA

## Abstract

In this paper, the fractional-order generalized Laguerre operational matrices (FGLOM) of fractional derivatives and fractional integration are derived. These operational matrices are used together with spectral tau method for solving linear fractional differential equations (FDEs) of order *ν* (0 < *ν* < 1) on the half line. An upper bound of the absolute errors is obtained for the approximate and exact solutions. Fractional-order generalized Laguerre pseudo-spectral approximation is investigated for solving nonlinear initial value problem of fractional order *ν*. The extension of the fractional-order generalized Laguerre pseudo-spectral method is given to solve systems of FDEs. We present the advantages of using the spectral schemes based on fractional-order generalized Laguerre functions and compare them with other methods. Several numerical examples are implemented for FDEs and systems of FDEs including linear and nonlinear terms. We demonstrate the high accuracy and the efficiency of the proposed techniques.

## Introduction

FDEs describe accurately many models in science and engineering such as bioengineering applications, porous or fractured media, electrochemical processes, viscoelastic materials [1–7]. Indeed most of FDEs do not have exact solutions. Therefore, there have been great attempts to develop numerical methods to solve them. Several analytical and numerical techniques for solving FDEs are proposed in [8–19].

Spectral methods are efficient techniques for solving differential equations accurately see for instance [20–27]. Bhrawy and Abdelkawy [4] proposed the formulation of Jacobi pseudospectral scheme for solving multi-dimensional fractional Schrodinger equations subject to different boundary conditions. The operational matrices for fractional variable-order of the derivative and integral of Jacobi polynomials were derived and used based on Jacobi tau scheme to solve the variable-order FDEs [28]. Recently, new accurate Petrov-Galerkin spectral solutions for FDEs are developed and analyzed in [29]. Moreover, spectral pseudospectral technique was investigated in [30] to approximate the solution of fractional integro-differential equation.

In the context of numerical methods for solving differential equations in the half-line, the first attempts to use Laguerre polynomials in the implementation of spectral methods to solve differential equations was the work of Gottlieb and Orszag [31]. After that, a series of published papers have appeared describing a range of various spectral methods based on Laguerre basis functions. Mikhailenko [32] developed an efficient algorithm based on the spectral Laguerre approximations of temporal derivatives for time-dependent problems. The authors of [33] proposed a new orthogonal family of generalized Laguerre functions to approximate the solution of differential equations of degenerate type. Xiao-Yong and Yan [34] investigated a pseudospectral scheme based on a class of modified generalized Laguerre to introduce a very efficient method for solving second-order differential equation in a long-time interval. Gulsu et al. [35] presented the Laguerre collocation method for solving a class of delay difference equations. Tatari and Haghighi [36] proposed an efficient mixed spectral collocation scheme to solve initial-boundary value problems in which Legendre and generalized Laguerre polynomials were used to discretize space and time variables.

On the other hand, results on numerical methods for FDEs seem to be lacking in the literature. In recent years, some authors have presented the generalized and modified generalized Laguerre spectral tau and collocation techniques for solving several types of linear and nonlinear FDEs on the half-line, (see [37, 38] and the references therein). However, it is also a very important task to develop the spectral techniques to obtain highly accurate solutions of FDEs on the half-line. Therefore, we present in this article a new family of orthogonal functions defined on the half-line namely, fractional-order generalized Laguerre functions.

In the present paper, we aim to construct the fractional-order generalized Laguerre operational matrices, of fractional derivative and integration, which are used to produce two efficient fractional-order generalized Laguerre tau schemes for solving numerically linear FDEs with initial conditions. We also aim to propose a new fractional-order generalized Laguerre collocation (FGLC) scheme for approximating the solution FDE of order *ν* (0 < *ν* < 1) with nonlinear terms. This approach is based on the operational matrix of fractional derivatives of these new functions, in which the nonlinear FDE is collocated at the *N* zeros of the fractional-order generalized Laguerre functions (FGLFs) defined on the interval (0, ∞). The resulting algebraic equations plus one algebraic equation (obtained from the initial condition), constitute (*N*+1) nonlinear algebraic equations. These equations may be solved by the Newton’s iterative technique to find the unknown fractional-order generalized Laguerre functions coefficients. We extend the application of FGLC method based on FGLFs to solve a system of linear FDEs with fractional orders less than 1. Several numerical examples are implemented to confirm the high accuracy and effectiveness of the new methods for solving FDES of fractional order *ν* (0 < *ν* < 1).

The remainder of this paper is organized as follows: we start by presenting some necessary definitions of the fractional calculus theory. In Section 3, we define the fractional-order generalized Laguerre functions. Section 4 is devoted to derive the main theorem of the paper which provides explicitly an operational matrix of fractional-order derivatives of the FGLFs. In Section 5, we derive an operational matrix of fractional-order integrals of the FGLFs. In Section 6, we apply the spectral methods based on the derived operational matrices FGLFs for solving FDEs and systems of FDEs including linear and nonlinear terms of fractional order less than 1. Several examples to illustrate the main ideas of this work are presented in Section 7. Finally Section 8 outlines the main conclusions.

## Preliminaries and Notations

We start this section by reviewing some definitions of fractional derivatives and integrals which will be employed in the sequel.

**Definition 2.1.** The Riemann-Liouville integral *J* ^*ν*^ *f*(*x*) and the Riemann-Liouville fractional derivative *D* ^*ν*^ *f*(*x*) of order *ν* > 0 are defined by

$$\begin{array}{ccc} J^{\nu }f\left(x\right) & = & \frac{1}{\Gamma (\nu )}\;\int_{0}^{x}{\left(x-t\right)}^{\nu -1}f\left(t\right)dt,\;x>0,\\ J^{0}f\left(x\right) & = & f(x), \end{array}$$ (1)

and

$$\begin{array}{c} D^{\nu }f\left(x\right)=J^{m-\nu }D^{m}f\left(x\right)=\frac{1}{\Gamma (m-\nu )}\int_{0}^{x}{\left(x-t\right)}^{m-\nu -1}\frac{d^{m}}{dt^{m}}f\left(t\right)dt,\;x>0, \end{array}$$ (2)

respectively, where *m*−1 < *ν* ≤ *m*, *m* ∈ *N* ^+^ and Γ(.) denotes the Gamma function.

**Definition 2.2.** The Caputo fractional integral and derivative operator satisfies

$$\begin{array}{c} J^{\nu }x^{\beta }=\frac{\Gamma \left(\beta +1\right)}{\Gamma \left(\beta +1+\nu \right)}\;x^{\beta +\nu }, \end{array}$$ (3)

$$\begin{array}{c} D^{\nu }x^{\beta }=\{\begin{array}{cc} 0, & \text{for}\;\beta \in N_{0}\;\text{and}\;\beta <\left\lceil \nu \right\rceil ,\\ \frac{\Gamma \left(\beta +1\right)}{\Gamma \left(\beta +1-\nu \right)}\;x^{\beta -\nu }, & \text{for}\;\beta \in N_{0}\;\text{and}\;\beta \geq \left\lceil \nu \right\rceil \;\text{or}\;\beta \notin N\;\text{and}\;\beta >\left\lfloor \nu \right\rfloor , \end{array} \end{array}$$ (4)

where ⌊*ν*⌋ and ⌈*ν*⌉ are the floor and ceiling functions respectively, while *N* = {1, 2, …} and *N*_0_ = {0, 1, 2, …}.

The Caputo’s fractional differentiation is a linear operation,

$$\begin{array}{c} D^{\nu }\left(\lambda f\left(x\right)+\mu g\left(x\right)\right)=\lambda D^{\nu }f\left(x\right)+\mu D^{\nu }g\left(x\right), \end{array}$$ (5)

where *λ* and *μ* are constants.

If *m*−1 < *ν* ≤ *m*, *m* ∈ *N*, then

$$\begin{array}{c} D^{\nu }J^{\nu }f\left(x\right)=f\left(x\right),\;J^{\nu }D^{\nu }f\left(x\right)=f\left(x\right)-\sum_{i=0}^{m-1}f^{\left(i\right)}\left(0^{+}\right)\frac{x^{i}}{i!},\;x>0. \end{array}$$ (6)

### Convert multi-order FDE into a system of FDE

Consider the multi-order FDE

$$\begin{array}{c} D^{\nu }u\left(x\right)=f\left(x,u\left(x\right),D^{\delta_{1}}u\left(x\right),\ldots ,D^{\delta_{n}}u\left(x\right)\right),u^{\left(k\right)}\left(0\right)=c_{k},\;k=0,1,\ldots ,m, \end{array}$$ (7)

where *m* < *ν* ≤ *m*+1, 0 < *δ*_1_ < *δ*_2_ < … ≤ *δ*_*n*_ < *ν*. This equation may be converted to a system of FDEs, as follows. Let *u*_1_ = *u* and assume

$$\begin{array}{c} D^{\delta_{1}}u_{1}=u_{2}. \end{array}$$ (8)

Case (i) If *m*−1 ≤ *δ*_1_ < *δ*_2_ ≤ *m*, then assume

$$\begin{array}{c} D^{\delta_{2}-\delta_{1}}u_{2}=u_{3}. \end{array}$$ (9)

Cases (ii) Consider *m*−1 ≤ *δ*_1_ < *m* ≤ *δ*_2_. If *δ*_1_ = *m*−1, then assume $D^{\delta_{2}-\delta_{1}}u_{2}=u_{3}$. If *m*−1 < *δ*_1_ < *m* ≤ *δ*_2_, then assume

$$\begin{array}{c} D^{m-\delta_{1}}u_{2}=u_{3}. \end{array}$$ (10)

similar steps can be converted the initial value problem Eq (7) to a system of FDE.

## Fractional-Order Generalized Laguerre Functions

We recall below some relevant properties of the generalized Laguerre polynomials (Szegö [39] and Funaro [40]). Let Λ = (0, ∞) and *w* ^(*α*)^(*x*) = *x* ^*α*^ *e* ^−*x*^ be a weight function on Λ. Consider the following inner product and norm

$$\begin{array}{c} {\left(u,v\right)}_{w^{\left(\alpha \right)}}=\int_{\Lambda }u\left(x\right)\;v\left(x\right)\;w^{\left(\alpha \right)}\left(x\right)\;dx,\;{\left||v|\right|}_{w^{\left(\alpha \right)}}={\left(u,v\right)}_{w^{\left(\alpha \right)}}^{\frac{1}{2}}. \end{array}$$

Next, let $L_{i}^{\left(\alpha \right)}\left(x\right)$ be the well-known generalized Laguerre polynomials. We know from [39] that for *α* > −1,

$$\begin{array}{c} L_{i+1}^{\left(\alpha \right)}\left(x\right)=\frac{1}{i+1}\left[\left(2i+\alpha +1-x\right)L_{i}^{\left(\alpha \right)}\left(x\right)-\left(i+\alpha \right)L_{i-1}^{\left(\alpha \right)}\left(x\right)\right],\;i=1,2,\ldots , \end{array}$$ (11)

where $L_{0}^{\left(\alpha \right)}\left(x\right)=1$ and $L_{1}^{\left(\alpha \right)}\left(x\right)=1+\alpha -x$.

The set of generalized Laguerre polynomials is a $L_{w^{\left(\alpha \right)}}^{2}(\Lambda )$-orthogonal system, thus

$$\begin{array}{c} \int_{0}^{\infty }L_{j}^{\left(\alpha \right)}\left(x\right)L_{k}^{\left(\alpha \right)}\left(x\right)w^{\left(\alpha \right)}\left(x\right)dx=h_{k}\delta_{jk}, \end{array}$$ (12)

where $h_{k}=\frac{\Gamma (k+\alpha +1)}{k!}$.

The analytical form of the generalized Laguerre polynomial on the interval Λ is given by

$$\begin{array}{c} L_{i}^{\left(\alpha \right)}\left(x\right)=\sum_{k=0}^{i}{\left(-1\right)}^{k}\frac{\Gamma (i+\alpha +1)}{\Gamma (k+\alpha +1)\;(i-k)!\;k!}\;x^{k},\;i=0,1,\ldots \end{array}$$ (13)

The special value

$$\begin{array}{c} D^{q}L_{i}^{\left(\alpha \right)}\left(0\right)={\left(-1\right)}^{q}\sum_{j=0}^{i-q}\frac{(i-j-1)!}{(q-1)!(i-j-q)!}L_{j}^{\left(\alpha \right)}\left(0\right),\;i⩾q, \end{array}$$ (14)

where $L_{j}^{\left(\alpha \right)}\left(0\right)=\frac{\Gamma (j+\alpha +1)}{\Gamma (\alpha +1)j!}$, will be of important use later.

Various kind of Laguerre polynomials/functions are used extensively in approximation theory and numerical analysis, for the interested reader see, [41–46], and the references therein.

### Definition of FGLFs

Now, we define a new fractional orthogonal functions based on generalized Laguerre polynomials to obtain the solution of FDEs more accurately. The FGLFs may be given by considering the change of variable *t* = *x* ^*λ*^ and *λ* > 0 on generalized Laguerre polynomials. Let the FGLFs $L_{i}^{\left(\alpha \right)}\left(x^{\lambda }\right)$ be denoted by $L_{i}^{\left(\alpha ,\lambda \right)}\left(x\right)$, thanks to Eq (11), then $L_{i}^{\left(\alpha ,\lambda \right)}\left(x\right)$ can be obtained from

$$\begin{array}{c} L_{i+1}^{\left(\alpha ,\lambda \right)}\left(x\right)=\frac{1}{i+1}\left[\left(2i+\alpha +1-x^{\lambda }\right)L_{i}^{\left(\alpha ,\lambda \right)}\left(x\right)-\left(i+\alpha \right)L_{i-1}^{\left(\alpha ,\lambda \right)}\left(x\right)\right],\;i=1,2,\ldots , \end{array}$$ (15)

where $L_{0}^{\left(\alpha ,\lambda \right)}\left(x\right)=1$ and $L_{1}^{\left(\alpha ,\lambda \right)}\left(x\right)=1+\alpha -x^{\lambda }$.

According to Eq (13), the analytic form of $L_{i}^{\left(\alpha ,\lambda \right)}\left(x\right)$ of degree *iλ* is given by

$$\begin{array}{c} L_{i}^{\left(\alpha ,\lambda \right)}\left(x\right)=\sum_{k=0}^{i}{\left(-1\right)}^{k}\frac{\Gamma (i+\alpha +1)}{\Gamma (k+\alpha +1)\;(i-k)!\;k!}\;x^{\lambda k},\;i=0,1,\ldots \end{array}$$ (16)

**Lemma 3.1** *The set of fractional-order generalized Laguerre functions is the* $L_{w^{\left(\alpha ,\lambda \right)}}^{2}(\Lambda )$-*orthogonal system*,

$$\begin{array}{c} \int_{0}^{\infty }L_{j}^{\left(\alpha ,\lambda \right)}\left(x\right)L_{k}^{\left(\alpha ,\lambda \right)}\left(x\right)w^{\left(\alpha ,\lambda \right)}\left(x\right)dx=h_{k}, \end{array}$$ (17)

*where* *w* ^(*α*, *λ*)^(*x*) = *λ* *x* ^(*α*+1)*λ*−1^ *e* ^−*x*^*λ*^^ *and* $h_{k}=\left\{ \begin{array}{cc} \frac{\Gamma (k+\alpha +1)}{k!},\; & j=k,\\ 0,\; & j\neq k. \end{array}\right.$

**Proof**. The proof of this lemma can be accomplished directly by using the definition of FGLFs and the orthogonality property of generalized Laguerre polynomials.

### The approximation of functions

Let $u(x)\in L_{w^{\left(\alpha ,\lambda \right)}}^{2}(\Lambda )$, then *u*(*x*) may be expressed in terms of FGLFs as

$$\begin{array}{ccc} u\left(x\right) & = & \sum_{j=0}^{\infty }c_{j}L_{j}^{\left(\alpha ,\lambda \right)}\left(x\right),\\ c_{j} & = & \frac{1}{h_{k}}\int_{0}^{\infty }u\left(x\right)L_{j}^{\left(\alpha ,\lambda \right)}\left(x\right)w^{\left(\alpha ,\lambda \right)}\left(x\right)dx,\;j=0,1,2,\cdots . \end{array}$$ (18)

In practice, only the first (*N*+1)-terms fractional-order generalized Laguerre functions are considered. Then we have

$$\begin{array}{c} u_{N}\left(x\right)=\sum_{j=0}^{N}c_{j}L_{j}^{\left(\alpha ,\lambda \right)}\left(x\right)=C^{T}\phi \left(x\right). \end{array}$$ (19)

where the fractional-order generalized Laguerre coefficient vector *C* and the fractional-order generalized Laguerre vector *ϕ*(*x*) are given respectively by

$$\begin{array}{ccc} C^{T} & = & [c_{0},c_{1},\ldots ,c_{N}],\\ \phi (x) & = & {\left[L_{0}^{\left(\alpha ,\lambda \right)}\left(x\right),L_{1}^{\left(\alpha ,\lambda \right)}\left(x\right),\ldots ,L_{N}^{\left(\alpha ,\lambda \right)}\left(x\right)\right]}^{T}. \end{array}$$ (20)

**Definition 3.1** *(Generalized Taylor’s formula). Suppose that* *D* ^*kλ*^ *u*(*x*) ∈ *C*[0, *L*] *for* *k* = 0, 1, …, *N*, *then we have*

$$\begin{array}{c} u\left(x\right)=\sum_{k=0}^{N}\frac{x^{k\lambda }}{\Gamma (k\lambda +1)}D^{k\nu }u\left(0^{+}\right)+\frac{x^{(N+1)\lambda }}{\Gamma ((N+1)\lambda +1)}D^{(N+1)\lambda }u\left(\eta \right), \end{array}$$

*where* 0 < *η* ≤ *x*, ∀*x* ∈ [0, *L*], *Also, one has*

$$\begin{array}{c} |u\left(x\right)-\sum_{k=0}^{N}\frac{x^{k\lambda }}{\Gamma (k\lambda +1)}D^{k\lambda }u\left(0^{+}\right)|\leq E_{\lambda }\frac{x^{(N+1)\lambda }}{\Gamma ((N+1)\lambda +1)}, \end{array}$$

*where* *E*_*λ*_ ≥ |*D* ^(*N*+1)*λ*^ *u*(*η*)|. *In case of* *λ* = 1, *the generalized Taylor’s formula is the classical Taylors formula*.

Now, the following Theorem presents an upper bound for estimating the error based on the expansion in terms of FGLFs.

**Theorem 3.2** *Suppose that* *D* ^*kλ*^ *u*(*x*) ∈ *C*[0, *L*] *for* *k* = 0, 1, …, *N*, (3+2*N*+*α*) > 0 *and* ${\mathbb{P}}_{N}^{\left(\alpha ,\lambda \right)}=Span\left\{L_{0}^{\left(\alpha ,\lambda \right)}(x),\cdots ,L_{N}^{\left(\alpha ,\lambda \right)}(x)\right\}.$ *If* *u*_*N*_(*x*) = *C* ^*T*^ *ϕ*(*x*) *is the best approximation to* *u*(*x*) *from* ${\mathbb{P}}_{N}^{\left(\alpha ,\lambda \right)}$, *then the error bound is presented as follows*

$$\begin{array}{c} {∥u\left(x\right)-u_{N}\left(x\right)∥}_{w^{\left(\alpha ,\lambda \right)}}\leq \frac{\sqrt{\Gamma (3+2N+\alpha )}\;E_{\lambda }}{\Gamma (N\lambda +1)}, \end{array}$$

*where* *E*_*λ*_ ≥ |*D* ^(*N*+1)*λ*^ *u*(*x*)|, *x* ∈ [0, *L*].

**Proof**. Considering the generalized Taylors formula

$$\begin{array}{c} u\left(x\right)=\sum_{k=0}^{N}\frac{x^{k\lambda }}{\Gamma (k\lambda +1)}D^{k\nu }u\left(0^{+}\right)+\frac{x^{(N+1)\lambda }}{\Gamma ((N+1)\lambda +1)}D^{(N+1)\lambda }u\left(\eta \right), \end{array}$$

where 0 < *η* ≤ *x*, ∀*x* ∈ [0, *L*], making use of Definition 3.1, we obtain

$$\begin{array}{c} |u\left(x\right)-\sum_{k=0}^{N}\frac{x^{k\lambda }}{\Gamma (k\lambda +1)}D^{k\lambda }u\left(0^{+}\right)|\leq E_{\lambda }\frac{x^{(N+1)\lambda }}{\Gamma ((N+1)\lambda +1)}. \end{array}$$

Since *u*_*N*_(*x*) = *C* ^*T*^ *ϕ*(*x*) is the best approximation to *u*(*x*) from ${\mathbb{P}}_{N}^{\left(\alpha ,\lambda \right)}$, then by the definition of the best approximation, we have

$$\begin{array}{c} \forall v_{N}\left(x\right)\in {\mathbb{P}}_{N}^{\left(\alpha ,\lambda \right)},\;{∥u\left(x\right)-u_{N}\left(x\right)∥}_{w^{\left(\alpha ,\lambda \right)}}\leq {∥u\left(x\right)-v_{N}\left(x\right)∥}_{w^{\left(\alpha ,\lambda \right)}}. \end{array}$$

It turns out that the previous inequality is also true if

$$\begin{array}{c} v_{N}\left(x\right)=\sum_{k=0}^{N}\frac{x^{k\lambda }}{\Gamma (k\lambda +1)}D^{k\lambda }u\left(0^{+}\right)\in {\mathbb{P}}_{N}^{\left(\alpha ,\lambda \right)}. \end{array}$$

Accordingly, we obtain

$$\begin{array}{ccc} {∥u\left(x\right)-u_{N}\left(x\right)∥}_{w^{\left(\alpha ,\lambda \right)}}^{2} & \leq & {∥u\left(x\right)-\sum_{k=0}^{N}\frac{x^{k\lambda }}{\Gamma (k\lambda +1)}D^{k\lambda }u\left(0^{+}\right)∥}_{w^{\left(\alpha ,\lambda \right)}}^{2}\\ & \leq & \frac{\lambda E_{\lambda }^{2}}{{\Gamma ((N+1)\lambda +1)}^{2}}\int_{0}^{\infty }x^{2(N+1)\lambda }x^{(\alpha +1)\lambda -1}e^{-x^{\lambda }}dx\\ & \leq & \frac{E_{\lambda }^{2}\;\Gamma \left(3+2N+\alpha \right)}{\Gamma {\left(N\lambda +1\right)}^{2}}. \end{array}$$ (21)

Now by taking the square roots, the theorem can be proved. Hence, an upper bound of the absolute errors is obtained for the approximate and exact solutions.

## Fractional-Order Generalized Laguerre Operational Matrix of Fractional Derivatives

Let $u(x)\in L_{w^{\left(\alpha ,\lambda \right)}}^{2}(\Lambda )$, then *u*(*x*) may be expressed in terms of fractional-order generalized Laguerre functions as

$$\begin{array}{ccc} u\left(x\right) & = & \sum_{j=0}^{\infty }a_{j}L_{j}^{\left(\alpha ,\lambda \right)}\left(x\right),\\ a_{j} & = & \frac{1}{h_{k}}\int_{0}^{\infty }u\left(x\right)L_{j}^{\left(\alpha ,\lambda \right)}\left(x\right)w^{\left(\alpha ,\lambda \right)}\left(x\right)dx,\;j=0,1,2,\cdots . \end{array}$$ (22)

In practice, only the first (*N*+1)-terms fractional-order generalized Laguerre functions are considered. Then we have

$$\begin{array}{c} u_{N}\left(x\right)=\sum_{j=0}^{N}a_{j}L_{j}^{\left(\alpha ,\lambda \right)}\left(x\right)=C^{T}\phi \left(x\right). \end{array}$$ (23)

where the fractional-order generalized Laguerre coefficient vector *C* and the fractional-order generalized Laguerre vector *ϕ*(*x*) are given respectively by

$$\begin{array}{ccc} C^{T} & = & [c_{0},c_{1},\ldots ,c_{N}],\\ \phi (x) & = & {\left[L_{0}^{\left(\alpha ,\lambda \right)}\left(x\right),L_{1}^{\left(\alpha ,\lambda \right)}\left(x\right),\ldots ,L_{N}^{\left(\alpha ,\lambda \right)}\left(x\right)\right]}^{T}, \end{array}$$ (24)

then the derivative of the vector *ϕ*(*x*) can be expressed by

$$\begin{array}{c} \frac{d\phi (x)}{dx}=D^{\left(1\right)}\phi \left(x\right), \end{array}$$ (25)

where **D** ^(1)^ is the (*N*+1)×(*N*+1) operational matrix of first-order derivative. If we define the *q* times repeated differentiation of fractional-order generalized Laguerre vector *ϕ*(*x*) by *D* ^*q*^ *ϕ*(*x*).

$$\begin{array}{c} D^{q}\phi \left(x\right)≃D^{\left(q\right)}\phi \left(x\right), \end{array}$$ (26)

where *q* is an integer value and **D** ^(*q*)^ is the operational matrix of differentiation of *ϕ*(*x*).

**Theorem 4.1** *Let* *ϕ*(*x*) *be fractional-order generalized Laguerre vector defined in* Eq (24) *and also suppose* 0 < *ν* < 1 *then*

$$\begin{array}{c} D^{\nu }\phi \left(x\right)≃D^{\left(\nu \right)}\phi \left(x\right), \end{array}$$ (27)

*where* ***D*** ^(*ν*)^ *is the* (*N*+1) × (*N*+1) *operational matrix of fractional derivative of order* *ν* *in the Caputo sense and is defined as follows:*

$$\begin{array}{c} D^{\left(\nu \right)}=(\begin{array}{ccccc} 0 & 0 & 0 & \ldots & 0\\ S_{\nu }\left(1,0,\lambda \right) & S_{\nu }\left(1,1,\lambda \right) & S_{\nu }\left(1,2,\lambda \right) & \ldots & S_{\nu }\left(1,N,\lambda \right)\\ \vdots & \vdots & \vdots & \ldots & \vdots \\ S_{\nu }\left(i,0,\lambda \right) & S_{\nu }\left(i,1,\lambda \right) & S_{\nu }\left(i,2,\lambda \right) & \ldots & S_{\nu }\left(i,N,\lambda \right)\\ \vdots & \vdots & \vdots & \ldots & \vdots \\ S_{\nu }\left(N,0,\lambda \right) & S_{\nu }\left(N,1,\lambda \right) & S_{\nu }\left(N,2,\lambda \right) & \ldots & S_{\nu }\left(N,N,\lambda \right) \end{array}) \end{array}$$ (28)

*where*

$$\begin{array}{c} S_{\nu }\left(i,j,\lambda \right)=\sum_{k=1}^{i}\sum_{s=0}^{j}\frac{{\left(-1\right)}^{k+s}\;j!\;\Gamma \left(i+\alpha +1\right)\;\Gamma \left(\lambda k+1\right)\;\Gamma \left(k-\frac{\nu }{\lambda }+\alpha +s+1\right)}{s!\;k!\;(i-k)!\;(j-s)!\;\Gamma (\lambda k-\nu +1)\;\Gamma (k+\alpha +1)\;\Gamma (\alpha +s+1)}. \end{array}$$

**Proof**. The analytic form of the fractional-order generalized Laguerre functions $L_{i}^{\left(\alpha ,\lambda \right)}\left(x\right)$ of degree *i* is given by Eq (16), Using Eqs (4), (5) and (16) we have

$$\begin{array}{ccc} D^{\nu }L_{i}^{\left(\alpha ,\lambda \right)}\left(x\right) & = & \sum_{k=0}^{i}\;{\left(-1\right)}^{k}\;\frac{\Gamma (i+\alpha +1)\;}{\left(i-k)!\;k!\;\Gamma (k+\alpha +1\right)}\;D^{\nu }x^{\lambda k}\\ & = & \sum_{k=1}^{i}{\left(-1\right)}^{k}\;\frac{\Gamma (i+\alpha +1)\Gamma (\lambda k+1)}{\left(i-k)!\;k!\;\Gamma (\lambda k-\nu +1)\;\Gamma (k+\alpha +1\right)}\;x^{\lambda k-\nu },\;i=1,\ldots ,N. \end{array}$$ (29)

Now, approximate *x* ^*λk*−*ν*^ by *N*+1 terms of fractional generalized Laguerre series yields

$$\begin{array}{c} x^{\lambda k-\nu }=\sum_{j=0}^{N}b_{j}L_{j}^{\left(\alpha ,\lambda \right)}\left(x\right), \end{array}$$ (30)

where *b*_*j*_ is given from Eq (22) with *u*(*x*) = *x* ^*λk*−*ν*^, and

$$\begin{array}{c} b_{j}=\sum_{s=0}^{j}\;{\left(-1\right)}^{s}\;\frac{j!\;\Gamma (k-\frac{\nu }{\lambda }+\alpha +s+1)\;}{\left(j-s)!\;(s)!\;\Gamma (s+\alpha +1\right)}, \end{array}$$ (31)

Employing Eqs (29)–(31) we get

$$\begin{array}{c} D^{\nu }L_{i}^{\left(\alpha ,\lambda \right)}\left(x\right)=\sum_{j=0}^{N}S_{\nu }\left(i,j,\lambda \right)L_{j}^{\left(\alpha ,\lambda \right)}\left(x\right),\;i=1,\cdots ,N, \end{array}$$ (32)

where

$$\begin{array}{c} S_{\nu }\left(i,j,\lambda \right)=\sum_{k=1}^{i}\sum_{s=0}^{j}\frac{{\left(-1\right)}^{k+s}\;j!\;\Gamma \left(i+\alpha +1\right)\;\Gamma \left(\lambda k+1\right)\;\Gamma \left(k-\frac{\nu }{\lambda }+\alpha +s+1\right)}{s!\;k!\;(i-k)!\;(j-s)!\;\Gamma (\lambda k-\nu +1)\;\Gamma (k+\alpha +1)\;\Gamma (\alpha +s+1)}. \end{array}$$

Accordingly, Eq (32) can be written in a vector form as follows:

$$\begin{array}{c} D^{\nu }L_{i}^{\left(\alpha ,\lambda \right)}\left(x\right)≃[S_{\nu }\left(i,0,\lambda \right),S_{\nu }\left(i,1,\lambda \right),S_{\nu }\left(i,2,\lambda \right),\ldots ,S_{\nu }\left(i,N,\lambda \right)]\phi \left(x\right),\;i=1,\ldots ,N. \end{array}$$ (33)

Eq (33) leads to the desired result.

## Fractional-Order Generalized Laguerre Operational Matrix of Fractional Integration

We aim to construct an operational matrix of fractional integration for fractional-order generalized Laguerre vector.

If *J* ^*q*^ *ϕ*(*x*) is the *q* (*q* is an integer value) times repeated integration of fractional-order generalized Laguerre vector *ϕ*(*x*), then

$$\begin{array}{c} J^{q}\phi \left(x\right)≃P^{\left(q\right)}\phi \left(x\right), \end{array}$$ (34)

where **P** ^(*q*)^ is the operational matrix of classical integration of *ϕ*(*x*).

**Theorem 5.1** *Let* *ϕ*(*x*) *be the fractional-order generalized Laguerre vector and* 0 < *ν* < 1 *then*

$$\begin{array}{c} J^{\nu }\phi \left(x\right)≃P^{\left(\nu \right)}\phi \left(x\right), \end{array}$$ (35)

*where* ***P*** ^(*ν*)^ *is the* (*N*+1) × (*N*+1) *operational matrix of fractional integration of order* *ν* *and* 0 < *ν* < 1 *in the Riemann-Liouville sense and is defined as follows:*

$$\begin{array}{c} P^{\left(\nu \right)}=(\begin{array}{ccccc} \Omega_{\nu }\left(0,0,\lambda \right) & \Omega_{\nu }\left(0,1,\lambda \right) & \Omega_{\nu }\left(0,2,\lambda \right) & \cdots & \Omega_{\nu }\left(0,N,\lambda \right)\\ \Omega_{\nu }\left(1,0,\lambda \right) & \Omega_{\nu }\left(1,1,\lambda \right) & \Omega_{\nu }\left(1,2,\lambda \right) & \cdots & \Omega_{\nu }\left(1,N,\lambda \right)\\ \vdots & \vdots & \vdots & \cdots & \vdots \\ \Omega_{\nu }\left(i,0,\lambda \right) & \Omega_{\nu }\left(i,1,\lambda \right) & \Omega_{\nu }\left(i,2,\lambda \right) & \cdots & \Omega_{\nu }\left(i,N,\lambda \right)\\ \vdots & \vdots & \vdots & \cdots & \vdots \\ \Omega_{\nu }\left(N,0,\lambda \right) & \Omega_{\nu }\left(N,1,\lambda \right) & \Omega_{\nu }\left(N,2,\lambda \right) & \cdots & \Omega_{\nu }\left(N,N,\lambda \right) \end{array}) \end{array}$$ (36)

*and*

$$\begin{array}{ccc} \Omega_{\nu }\left(i,j,\lambda \right) & = & \sum_{k=0}^{i}\frac{{\left(-1\right)}^{k}\;\Gamma \left(i+\alpha +1\right)\;j!\;\Gamma \left(k\lambda +1\right)}{\Gamma \left(k+\alpha +1\right)\;(i-k)!\;k!\;\Gamma (k\lambda +\nu +1)}\\ & \times & \sum_{r=0}^{j}\frac{{\left(-1\right)}^{r}\;\Gamma \left(r+k+\frac{\nu }{\lambda }+\alpha +1\right)}{(j-r)!\;r!\;\Gamma \left(r+\alpha +1\right)}. \end{array}$$ (37)

**Proof**. From Eqs (16) and (3), we have

$$\begin{array}{ccc} J^{\nu }L_{i}^{\left(\alpha ,\lambda \right)}\left(x\right) & = & \sum_{k=0}^{i}{\left(-1\right)}^{k}\;\frac{\Gamma (i+\alpha +1)\;}{\left(i-k)!\;k!\;\Gamma (k+\alpha +1\right)}\;J^{\nu }x^{k\lambda }\\ & = & \sum_{k=0}^{i}{\left(-1\right)}^{k}\;\frac{\Gamma (i+\alpha +1)\;\Gamma (k\lambda +1)}{\left(i-k)!\;k!\;\Gamma (k\lambda +\nu +1)\;\Gamma (k+\alpha +1\right)}\;x^{k\lambda +\nu },\;i=0,1,\cdots ,N. \end{array}$$ (38)

The approximation of *x* ^*kλ*+*ν*^ using *N*+1 terms of fractional-order generalized Laguerre series, yields

$$\begin{array}{c} x^{k\lambda +\nu }=\sum_{j=0}^{N}c_{j}L_{j}^{\left(\alpha ,\lambda \right)}\left(x\right), \end{array}$$ (39)

where *c*_*j*_ is given from Eq (22) with *u*(*x*) = *x* ^*kλ*+*ν*^, that is

$$\begin{array}{c} c_{j}=\sum_{r=0}^{j}{\left(-1\right)}^{r}\;\frac{j!\;\Gamma (r+k+\frac{\nu }{\lambda }+\alpha +1)\;}{\left(j-r)!\;r!\;\Gamma (r+\alpha +1\right)},\;j=1,2,\cdots ,N. \end{array}$$ (40)

Thanks to Eqs (38) and (39), gives

$$\begin{array}{c} J^{\nu }L_{i}^{\left(\alpha ,\lambda \right)}\left(x\right)=\sum_{j=0}^{N}\Omega_{\nu }\left(i,j\right)L_{j}^{\left(\alpha ,\lambda \right)}\left(x\right),\;i=0,1,\cdots ,N, \end{array}$$ (41)

where

$$\begin{array}{ccc} \Omega_{\nu }\left(i,j,\lambda \right) & = & \sum_{k=0}^{i}\frac{{\left(-1\right)}^{k}\;\Gamma \left(i+\alpha +1\right)\;j!\;\Gamma \left(k\lambda +1\right)}{\Gamma \left(k+\alpha +1\right)\;(i-k)!\;k!\;\Gamma (k\lambda +\nu +1)}\\ & \times & \sum_{r=0}^{j}\frac{{\left(-1\right)}^{r}\;\Gamma \left(r+k+\frac{\nu }{\lambda }+\alpha +1\right)}{(j-r)!\;r!\;\Gamma \left(r+\alpha +1\right)}\;j=1,2,\cdots N. \end{array}$$

The vector form of Eq (41) is

$$\begin{array}{c} J^{\nu }L_{i}^{\left(\alpha ,\lambda \right)}\left(x\right)≃[\Omega_{\nu }\left(i,0,\lambda \right),\Omega_{\nu }\left(i,1,\lambda \right),\Omega_{\nu }\left(i,2,\lambda \right),\cdots ,\Omega_{\nu }\left(i,N,\lambda \right)]\phi \left(x\right),\;i=0,1,\cdots ,N. \end{array}$$ (42)

Eq (42) leads to the desired result.

## Application of Fractional-Order Generalized Laguerre Operational Matrices for FDEs

The main aim of this section is to propose two different ways to approximate linear FDEs using the fractional-order generalized Laguerre tau method based on fractional-order Laguerre operational matrices of differentiation and integration such that it can be implemented efficiently. Also, we propose a new collocation method for solve nonlinear FDEs and systems of FDEs based on the fractional-order generalized Laguerre ffunctions.

### Operational matrix of fractional derivatives

A direct solution technique is proposed here, to solve linear FDEs using the fractional-order generalized Laguerre tau method in combination with FGLOM.

Let us consider the linear FDE

$$\begin{array}{c} D^{\nu }u\left(x\right)+\gamma u\left(x\right)=g\left(x\right),\;\text{in}\;\Lambda =\left(0,\infty \right), \end{array}$$ (43)

subject to

$$\begin{array}{c} u\left(0\right)=u_{0}, \end{array}$$ (44)

where *γ* is a real constant coefficient and also 0 < *ν* ≤ 1, while *D* ^*ν*^ *u*(*x*) ≡ *u* ^(*ν*)^(*x*) is the Caputo fractional derivative of order *ν*.

Now we will implement an efficient algorithm to solve the fractional initial value problem; Eqs (43)–(44). We approximate *u*(*x*) and *g*(*x*) by fractional-order generalized Laguerre polynomials as

$$\begin{array}{c} u\left(x\right)≃\sum_{i=0}^{N}c_{i}L_{i}^{\left(\alpha ,\lambda \right)}\left(x\right)=C^{T}\phi \left(x\right), \end{array}$$ (45)

$$\begin{array}{c} g\left(x\right)≃\sum_{i=0}^{N}g_{i}L_{i}^{\left(\alpha ,\lambda \right)}\left(x\right)=G^{T}\phi \left(x\right), \end{array}$$ (46)

where vector *G* = [*g*_0_, …, *g*_*N*_]^*T*^ is known and *C* = [*c*_0_, …, *c*_*N*_]^*T*^ is an unknown vector.

By using Theorem 4.1 (relation Eqs (27) and (45)) we have

$$\begin{array}{c} D^{\nu }u\left(x\right)≃C^{T}D^{\nu }\phi \left(x\right)=C^{T}D^{\left(\nu \right)}\phi \left(x\right), \end{array}$$ (47)

Employing Eqs (45)–(47), the residual *R*_*N*_(*x*) for Eq (43) can be written as

$$\begin{array}{c} R_{N}\left(x\right)=\left(C^{T}D^{\left(\nu \right)}+\gamma C^{T}-G^{T}\right)\phi \left(x\right). \end{array}$$ (48)

The application of spectral tau scheme, see [47], provides a system of (*N*) linear equations,

$$\begin{array}{c} \left\langle R_{N}\left(x\right),L_{j}^{\left(\alpha ,\lambda \right)}\left(x\right)\right\rangle =\int_{0}^{\infty }w^{\left(\alpha ,\lambda \right)}\left(x\right)R_{N}\left(x\right)L_{j}^{\left(\alpha ,\lambda \right)}\left(x\right)dx=0\;\;j=0,1,\ldots ,N. \end{array}$$ (49)

Substituting Eq (45) in Eq (44) yields

$$\begin{array}{c} u\left(0\right)=C^{T}D^{\left(0\right)}\phi \left(0\right)=u_{0}. \end{array}$$ (50)

The combination of Eqs (49) and (50) gives a system of algebraic equations, which may be solved by any direct solver technique to obtain the spectral solution *u*_*N*_(*x*).

### Operational matrix of fractional integration

Here, the fractional-order generalized Laguerre tau scheme in conjunction of the derived operational matrix is proposed for solving the linear FDEs. The basic steps of such scheme are: (i) The aforementioned fractional differential equation is converted into a fractional integrated form equation by making use of fractional integration for this equation. (ii) Subsequently, this integrated form equation is approximated by expressing the numerical solution as a linear combination of fractional-order generalized Laguerre functions. (iii) Finally, the problem is transformed into a system of algebraic equations by using the operational matrix of fractional integration of fractional-order generalized Laguerre functions.

In order to show the importance of FGLOM of fractional integration, we apply it to solve the following FDE:

$$\begin{array}{c} D^{\nu }u\left(x\right)+\gamma u\left(x\right)=f\left(x\right),\;\text{in}\;\Lambda =\left(0,\infty \right), \end{array}$$ (51)

with initial condition

$$\begin{array}{c} u\left(0\right)=u_{0}, \end{array}$$ (52)

where *γ* is a real constant coefficient and also 0 < *ν* ≤ 1. Moreover, *D* ^*ν*^ *u*(*x*) denotes the Caputo fractional derivative of order *ν* for *u*(*x*) and the value *u*_0_ describes the initial condition of *u*(*x*). If we apply the Riemann-Liouville integral of order *ν* on Eq (51) and after making use of Eq (6), we get the integrated form of Eq (51), namely

$$\begin{array}{c} u\left(x\right)-\sum_{j=0}^{m-1}u\left(0^{+}\right)\frac{x^{j}}{j!}+\gamma J^{\nu }u\left(x\right)=J^{\nu }f\left(x\right), \end{array}$$ (53)

this implies that

$$\begin{array}{c} u\left(x\right)+\gamma J^{\nu }u\left(x\right)=g\left(x\right), \end{array}$$ (54)

where

$$\begin{array}{c} g\left(x\right)=J^{\nu }f\left(x\right)+\sum_{j=0}^{m-1}u_{0}\frac{x^{j}}{j!}. \end{array}$$

Now, approximating *u*(*x*) and *g*(*x*) by employing the fractional-order generalized Laguerre functions as

$$\begin{array}{c} u_{N}\left(x\right)≃\sum_{i=0}^{N}c_{i}L_{i}^{\left(\alpha ,\lambda \right)}\left(x\right)=C^{T}\phi \left(x\right), \end{array}$$ (55)

$$\begin{array}{c} g\left(x\right)≃\sum_{i=0}^{N}g_{i}L_{i}^{\left(\alpha ,\lambda \right)}\left(x\right)=G^{T}\phi \left(x\right). \end{array}$$ (56)

In virtue of Theorem 5.1 (relation Eq (35)), the Riemann-Liouville integral of order *ν* of Eq (55), can be obtained from

$$\begin{array}{c} J^{\nu }u_{N}\left(x\right)≃C^{T}J^{\nu }\phi \left(x\right)≃C^{T}P^{\left(\nu \right)}\phi \left(x\right). \end{array}$$ (57)

Employing Eq (55) the residual *R*_*N*_(*x*) for Eq (54) can be written as

$$\begin{array}{c} R_{N}\left(x\right)=\left(C^{T}+\gamma C^{T}P^{\left(\nu \right)}-G^{T}\right)\phi \left(x\right). \end{array}$$ (58)

Finally, applying the spectral tau method to the residual gives

$$\begin{array}{c} {\left(R_{N}\left(x\right),L_{j}^{\left(\alpha ,\lambda \right)}\left(x\right)\right)}_{w^{\left(\alpha ,\lambda \right)}\left(x\right)}=\int_{0}^{\infty }R_{N}\left(x\right)\;w^{\left(\alpha ,\lambda \right)}\left(x\right)\;L_{j}^{\left(\alpha ,\lambda \right)}\left(x\right)dx=0,\;\;j=0,1,\cdots ,N. \end{array}$$ (59)

Also from Eq (55) into Eq (52) yields

$$\begin{array}{c} u\left(0\right)=C^{T}\phi \left(0\right)=u_{0}. \end{array}$$ (60)

Eqs (59) and (60) generate *N* of linear equations.

### Nonlinear initial FDEs

Regarding the nonlinear fractional initial value problems on the semi-infinite domain, we investigate the spectral fractional-order generalized Laguerre collocation FGLC scheme in combination with FGLOM of fractional derivative to obtain an accurate approximate solution *u*_*N*_(*x*). The problem is collocated at *N* nodes of the fractional-order generalized Laguerre-Gauss interpolation defined on Λ. The resulting equations along with the algebraic equation resumed form the initial condition consist an algebraic system of (*N*+1) equations which may be solved numerically by Newton’s iterative method.

Consider the nonlinear FDE

$$\begin{array}{c} D^{\nu }u\left(x\right)=F\left(x,u\left(x\right)\right),\;\text{in}\;\Lambda =\left(0,\infty \right), \end{array}$$ (61)

with initial conditions Eq (44), where *F* can be nonlinear in general.

In order to use FGLOM for this problem, we first expand *u*(*x*) and *D* ^*ν*^ *u*(*x*) as Eqs (45) and (47) respectively. By substituting these approximations into Eq (61) we have

$$\begin{array}{c} C^{T}D^{\left(\nu \right)}\phi \left(x\right)≃F\left(x,C^{T}\phi \left(x\right)\right). \end{array}$$ (62)

Substituting Eqs (45) and (26) into Eq (44), we obtain

$$\begin{array}{c} u\left(0\right)=C^{T}\phi \left(0\right)=u_{0}. \end{array}$$ (63)

Collocating Eq (62) at the zeros of the fractional-order Laguerre functions provides *N* equations together with one equation from Eq (63) consist a system of *N*+1 nonlinear equations. Consequently, the solution *u*_*N*_(*x*) may be archived by implementing Newton’s iterative scheme.

**Corollary 6.1** *In particular, the special case for generalized Laguerre polynomials may be obtained directly by taking* *λ* = 0 *in the fractional-order Laguerre functions, which are denoted by* $L_{i}^{\left(\alpha \right)}(x)$. *However, the classical Laguerre polynomials may be achieved by replacing* *λ* = 1 *and* *α* = 0, *which are used most frequently in practice and will simply be denoted by* *L*_*i*_(*x*).

### FGLC method for solving systems of FDEs

We use the FGLC method to numerically solve the general form of systems of nonlinear FDE, namely

$$\begin{array}{c} D^{\nu_{i}}u_{i}\left(x\right)=F_{i}(x,u_{1}\left(x\right),u_{2}\left(x\right),\ldots ,u_{n}\left(x\right)),\;x\in \Lambda ,\;i=1,\ldots ,n, \end{array}$$ (64)

with initial conditions

$$\begin{array}{c} u_{i}\left(0\right)=u_{i0},\;\;\;\;i=1,\ldots ,n, \end{array}$$ (65)

where 0 < *ν*_*i*_ ≤ 1.

Let

$$\begin{array}{c} u_{iN}\left(x\right)=\sum_{j=0}^{N}a_{ij}L_{j}^{\left(\alpha ,\lambda \right)}\left(x\right), \end{array}$$ (66)

The fractional derivatives $D^{\nu_{i}}u(x),$ can be expressed in terms of the expansion coefficients *a*_*ij*_ using Eq (27). The implementation of fractional generalized Laguerre collocation method to solve Eqs (64)–(65) is to find *u*_*iN*_(*x*) ∈ *Q*_*N*_(Λ) such that

$$\begin{array}{c} D^{\nu_{i}}u_{iN}\left(x\right)=F_{i}(x,u_{1N}\left(x\right),u_{2N}\left(x\right),...,u_{nN}\left(x\right)),\;x\in \Lambda , \end{array}$$ (67)

is satisfied exactly at the collocation points $x_{i,N,k}^{\left(\alpha ,\lambda \right)},k=0,1,\cdots ,N-1$, *i* = 1, ⋯, *n*, which immediately yields

$$\begin{array}{ccc} \sum_{j=0}^{N}a_{ij}D^{\nu_{i}}L_{j}^{\left(\alpha ,\lambda \right)}\left(x_{i,N,k}^{\left(\alpha ,\lambda \right)}\right) & = & F_{i}(x_{i,N,k}^{\left(\alpha ,\lambda \right)},\sum_{j=0}^{N}a_{1j}L_{j}^{\left(\alpha ,\lambda \right)}\left(x_{1,N,k}^{\left(\alpha ,\lambda \right)}\right),\sum_{j=0}^{N}a_{2j}L_{j}^{\left(\alpha ,\lambda \right)}\left(x_{2,N,k}^{\left(\alpha ,\lambda \right)}\right),\\ & & \ldots ,\sum_{j=0}^{N}a_{nj}L_{j}^{\left(\alpha ,\lambda \right)}\left(x_{n,N,k}^{\left(\alpha ,\lambda \right)}\right)), \end{array}$$ (68)

with Eq (65) written in the form

$$\begin{array}{c} \sum_{j=0}^{N}a_{ij}L_{j}^{\left(\alpha ,\lambda \right)}\left(0\right)=u_{i0},\;\;i=1,\cdots ,n. \end{array}$$ (69)

This means the system Eq (64) with its initial conditions have been reduced to a system of *n*(*N*+1) nonlinear algebraic Eqs (68)–(69), which may be solved by using any standard iteration technique.

## Illustrative Examples

We present in this section, several illustrative examples by implementing the proposed spectral algorithms in this article. These examples are chosen such that their exact solutions are known. The results for these examples demonstrate that the proposed methods are accurate, effective and convenient.

**Example 1** *Consider the equation*

$$\begin{array}{ccc} D^{\nu }u\left(x\right)+u\left(x\right) & = & \frac{\Gamma \left(3\right)}{\Gamma \left(3-\nu \right)}\;x^{2-\nu }+x^{2},\;\;0<\nu <1,\;x\in \Lambda , \end{array}$$

*the exact solution is given by* *u*(*x*) = *x* ^2^.

Now, we implement the spectral tau scheme based on the FGLOM of fractional derivative with *N* = 6, then the approximate solution can be expanded as

$$\begin{array}{c} u_{N}\left(x\right)=\sum_{i=0}^{N}c_{i}L_{i}^{\left(\alpha ,\lambda \right)}\left(x\right)=C^{T}\phi \left(x\right). \end{array}$$

If we choose $\lambda =\frac{1}{3}$ and $\nu =\frac{1}{3}$, then

$$\begin{array}{c} D^{\left(\nu \right)}=(\begin{array}{ccccc} 0 & 0 & 0 & \ldots & 0\\ S_{\nu }\left(1,0,\frac{1}{3}\right) & S_{\nu }\left(1,1,\frac{1}{3}\right) & S_{\nu }\left(1,2,\frac{1}{3}\right) & \ldots & S_{\nu }\left(1,6,\frac{1}{3}\right)\\ \vdots & \vdots & \vdots & \ldots & \vdots \\ S_{\nu }\left(i,0,\frac{1}{3}\right) & S_{\nu }\left(i,1,\frac{1}{3}\right) & S_{\nu }\left(i,2,\frac{1}{3}\right) & \ldots & S_{\nu }\left(i,6,\frac{1}{3}\right)\\ \vdots & \vdots & \vdots & \ldots & \vdots \\ S_{\nu }\left(6,0,\frac{1}{3}\right) & S_{\nu }\left(6,1,\frac{1}{3}\right) & S_{\nu }\left(6,2,\frac{1}{3}\right) & \ldots & S_{\nu }\left(6,6,\frac{1}{3}\right) \end{array}),\;G=(\begin{array}{c} g_{0}\\ g_{1}\\ g_{2}\\ \vdots \\ g_{6} \end{array}), \end{array}$$

where *g*_*j*_ and *S*_*ν*_(*i*, *j*, *λ*) are defined in Eqs (22) and (28).

Using Eq (49), we obtain

$$\begin{array}{ccc} c_{0}+S_{\nu }\left(1,0,\frac{1}{3}\right)c_{1}+S_{\nu }\left(2,0,\frac{1}{3}\right)c_{2}+S_{\nu }\left(3,0,\frac{1}{3}\right)c_{3}+S_{\nu }\left(4,0,\frac{1}{3}\right)c_{4}+S_{\nu }\left(5,0,\frac{1}{3}\right)c_{5}+S_{\nu }\left(6,0,\frac{1}{3}\right)c_{6} & = & g_{0},\\ c_{1}+S_{\nu }\left(1,1,\frac{1}{3}\right)c_{1}+S_{\nu }\left(2,1,\frac{1}{3}\right)c_{2}+S_{\nu }\left(3,1,\frac{1}{3}\right)c_{3}+S_{\nu }\left(4,1,\frac{1}{3}\right)c_{4}+S_{\nu }\left(5,1,\frac{1}{3}\right)c_{5}+S_{\nu }\left(6,1,\frac{1}{3}\right)c_{6} & = & g_{1},\\ c_{2}+S_{\nu }\left(1,2,\frac{1}{3}\right)c_{1}+S_{\nu }\left(2,2,\frac{1}{3}\right)c_{2}+S_{\nu }\left(3,2,\frac{1}{3}\right)c_{3}+S_{\nu }\left(4,2,\frac{1}{3}\right)c_{4}+S_{\nu }\left(5,2,\frac{1}{3}\right)c_{5}+S_{\nu }\left(6,2,\frac{1}{3}\right)c_{6} & = & g_{2},\\ c_{3}+S_{\nu }\left(1,3,\frac{1}{3}\right)c_{1}+S_{\nu }\left(2,3,\frac{1}{3}\right)c_{2}+S_{\nu }\left(3,3,\frac{1}{3}\right)c_{3}+S_{\nu }\left(4,3,\frac{1}{3}\right)c_{4}+S_{\nu }\left(5,3,\frac{1}{3}\right)c_{5}+S_{\nu }\left(6,3,\frac{1}{3}\right)c_{6} & = & g_{3},\\ c_{4}+S_{\nu }\left(1,4,\frac{1}{3}\right)c_{1}+S_{\nu }\left(2,4,\frac{1}{3}\right)c_{2}+S_{\nu }\left(3,4,\frac{1}{3}\right)c_{3}+S_{\nu }\left(4,4,\frac{1}{3}\right)c_{4}+S_{\nu }\left(5,4,\frac{1}{3}\right)c_{5}+S_{\nu }\left(6,4,\frac{1}{3}\right)c_{6} & = & g_{4},\\ c_{5}+S_{\nu }\left(1,5,\frac{1}{3}\right)c_{1}+S_{\nu }\left(2,5,\frac{1}{3}\right)c_{2}+S_{\nu }\left(3,5,\frac{1}{3}\right)c_{3}+S_{\nu }\left(4,5,\frac{1}{3}\right)c_{4}+S_{\nu }\left(5,5,\frac{1}{3}\right)c_{5}+S_{\nu }\left(6,5,\frac{1}{3}\right)c_{6} & = & g_{5}, \end{array}$$ (70)

The treatment of initial condition using Eq (44), yields

$$\begin{array}{ccc} & & c_{0}+\left(\alpha +1\right)c_{1}+\frac{\left(\alpha +1)(\alpha +2\right)}{2}c_{2}+\frac{\left(\alpha +1)(\alpha +2)(\alpha +3\right)}{6}c_{3}+\frac{\left(\alpha +1)(\alpha +2)(\alpha +3)(\alpha +4\right)}{24}c_{4}\\ & & \;+\frac{\left(\alpha +1)(\alpha +2)(\alpha +3)(\alpha +4)(\alpha +5\right)}{120}c_{5}+\frac{\left(\alpha +1)(\alpha +2)(\alpha +3)(\alpha +4)(\alpha +5)(\alpha +6\right)}{720}c_{6}=0 \end{array}$$ (71)

Solving the resulted system of algebraic Eqs (70)–(71) provides the unknown coefficients in terms of *α*.

Accordingly, the approximate solution can be written as

$$\begin{array}{c} u_{N}\left(x\right)=\sum_{i=0}^{6}c_{i}L_{i}^{\left(\alpha ,\frac{1}{3}\right)}\left(x\right)=x^{2}. \end{array}$$

Tables 1 and 2 list the values of *c*_0_, *c*_1_, *c*_2_, *c*_3_, *c*_4_, *c*_5_ and *c*_6_ with different choices of *α* and two choices of *ν* = 1/3 and *ν* = 1/4. Indeed, we can achieve the exact solution of this problem with all choices of the fractional-order generalized Laguerre parameter *α*.

**Table 1:** The values *c*_0_, *c*_1_, *c*_2_, … and *c*_6_ for different values of *α* at $\nu =\frac{1}{3}$ for Example 1.

*α*	*c*_0_	*c*_1_	*c*_2_	*c*_3_	*c*_4_	*c*_5_	*c*_6_
0	720	-4320	10800	-14400	10800	-4320	720
1	5040	-15120	25200	-25200	15120	-5040	720
2	20160	-40320	50400	-40320	20160	-5760	720
3	60480	-90720	90720	-60480	25920	-6480	720

**Table 2:** The values *c*_0_, *c*_1_, *c*_2_, … and *c*_6_ for different values of *α* at $\nu =\frac{1}{4}$ for Example 1.

*α*	*c*_0_	*c*_1_	*c*_2_	*c*_3_	*c*_4_	*c*_5_	*c*_6_
0	720	-4300	10800	-14000	10800	-4300	720
1	5000	-15000	2500	-25000	15000	-5000	720
2	20000	-40000	50000	-40000	20000	-6000	700
3	60000	-90000	90000	-60000	30000	-6000	700

**Example 2** *Consider the equation*

$$\begin{array}{c} D^{\nu }u\left(x\right)+u\left(x\right)=\frac{\Gamma \left(4\right)}{\Gamma \left(4-\nu \right)}\;x^{3-\nu }-\frac{\Gamma \left(2\right)}{\Gamma \left(2-\nu \right)}\;x^{1-\nu }+x^{3}-x,\;\;0<\nu <1,\;x\in \Lambda , \end{array}$$

*the exact solution is given by* *u*(*x*) = *x* ^3^−*x*.

If we apply the technique described in Section 6.2 based on the FGLOM of fractional integration with *N* = 6, then the approximate solution can be written as follows

$$\begin{array}{c} u_{N}\left(x\right)=\sum_{i=0}^{6}c_{i}L_{i}^{\left(\alpha ,\lambda \right)}\left(x\right)=C^{T}\phi \left(x\right), \end{array}$$

We put $\lambda =\frac{1}{2}$ and $\nu =\frac{1}{2}$, we have

$$\begin{array}{c} P^{\left(\frac{1}{2}\right)}=(\begin{array}{ccccc} \Omega_{\frac{1}{2}}\left(0,0,\frac{1}{2}\right) & \Omega_{\frac{1}{2}}\left(0,1,\frac{1}{2}\right) & \Omega_{\frac{1}{2}}\left(0,2,\frac{1}{2}\right) & \ldots & \Omega_{\frac{1}{2}}\left(0,6,\frac{1}{2}\right)\\ \vdots & \vdots & \vdots & \ldots & \vdots \\ \Omega_{\frac{1}{2}}\left(i,0,\frac{1}{2}\right) & \Omega_{\frac{1}{2}}\left(i,1,\frac{1}{2}\right) & \Omega_{\frac{1}{2}}\left(i,2,\frac{1}{2}\right) & \ldots & \Omega_{\frac{1}{2}}\left(i,6,\frac{1}{2}\right)\\ \vdots & \vdots & \vdots & \ldots & \vdots \\ \Omega_{\frac{1}{2}}\left(6,0,\frac{1}{2}\right) & \Omega_{\frac{1}{2}}\left(6,1,\frac{1}{2}\right) & \Omega_{\frac{1}{2}}\left(6,2,\frac{1}{2}\right) & \ldots & \Omega_{\frac{1}{2}}\left(6,6,\frac{1}{2}\right) \end{array}),\;G=(\begin{array}{c} g_{0}\\ g_{1}\\ g_{2}\\ \vdots \\ g_{6} \end{array}). \end{array}$$

where *g*_*j*_ and Ω_*ν*_(*i*, *j*, *λ*) are defined in Eqs (22) and (36).

Using Eq (59), we obtain the following:

$$\begin{array}{c} \left(1+\Omega_{\frac{1}{2}}\left(0,0,\frac{1}{2}\right)\right)c_{0}+\Omega_{\frac{1}{2}}\left(1,0,\frac{1}{2}\right)c_{1}+\Omega_{\frac{1}{2}}\left(2,0,\frac{1}{2}\right)c_{2}+\ldots +\Omega_{\frac{1}{2}}\left(6,0,\frac{1}{2}\right)c_{6}=g_{0},\\ \Omega_{\frac{1}{2}}\left(0,1,\frac{1}{2}\right)c_{0}+\left(1+\Omega_{\frac{1}{2}}\left(1,1,\frac{1}{2}\right)\right)c_{1}+\Omega_{\frac{1}{2}}\left(2,1,\frac{1}{2}\right)c_{2}+\ldots +\Omega_{\frac{1}{2}}\left(6,1,\frac{1}{2}\right)c_{6}=g_{1},\\ \Omega_{\frac{1}{2}}\left(0,2,\frac{1}{2}\right)c_{0}+\Omega_{\frac{1}{2}}\left(1,2,\frac{1}{2}\right)c_{1}+\left(1+\Omega_{\frac{1}{2}}\left(2,2,\frac{1}{2}\right)\right)c_{2}+\ldots +\Omega_{\frac{1}{2}}\left(6,2,\frac{1}{2}\right)c_{6}=g_{2},\\ \Omega_{\frac{1}{2}}\left(0,3,\frac{1}{2}\right)c_{0}+\ldots +\left(1+\Omega_{\frac{1}{2}}\left(3,3,\frac{1}{2}\right)\right)c_{3}+\Omega_{\frac{1}{2}}\left(4,3,\frac{1}{2}\right)c_{4}+\ldots +\Omega_{\frac{1}{2}}\left(6,3,\frac{1}{2}\right)c_{6}=g_{3},\\ \Omega_{\frac{1}{2}}\left(0,4,\frac{1}{2}\right)c_{0}+\ldots +\left(1+\Omega_{\frac{1}{2}}\left(3,4,\frac{1}{2}\right)\right)c_{4}+\Omega_{\frac{1}{2}}\left(5,4,\frac{1}{2}\right)c_{5}+\Omega_{\frac{1}{2}}\left(6,4,\frac{1}{2}\right)c_{6}=g_{4},\\ \Omega_{\frac{1}{2}}\left(0,5,\frac{1}{2}\right)c_{0}+\ldots +\Omega_{\frac{1}{2}}\left(4,5,\frac{1}{2}\right)c_{4}+\left(1+\Omega_{\frac{1}{2}}\left(5,5,\frac{1}{2}\right)\right)c_{5}+\Omega_{\frac{1}{2}}\left(6,5,\frac{1}{2}\right)c_{6}=g_{5}, \end{array}$$ (72)

with *ν* = 1/2. Now, by applying Eq (60), we have

$$\begin{array}{ccc} & & c_{0}+\left(\alpha +1\right)c_{1}+\frac{\left(\alpha +1)(\alpha +2\right)}{2}c_{2}+\frac{\left(\alpha +1)(\alpha +2)(\alpha +3\right)}{6}c_{3}+\frac{\left(\alpha +1)(\alpha +2)(\alpha +3)(\alpha +4\right)}{24}c_{4}\\ & & \;+\frac{\left(\alpha +1)(\alpha +2)(\alpha +3)(\alpha +4)(\alpha +5\right)}{120}c_{5}+\frac{\left(\alpha +1)(\alpha +2)(\alpha +3)(\alpha +4)(\alpha +5)(\alpha +6\right)}{720}c_{6}=0 \end{array}$$ (73)

Finally, solving the resulted system of algebraic Eqs (72)–(73) provides the unknown coefficients with $\nu =\frac{1}{2}$ and various choices of *α*.

Thus we can write

$$\begin{array}{c} u_{N}\left(x\right)=\sum_{i=0}^{6}c_{i}L_{i}^{\left(\alpha ,\frac{1}{2}\right)}\left(x\right)=x^{3}-x, \end{array}$$

Table 3 presents the values *c*_0_, *c*_1_, *c*_2_, … and *c*_6_ for several choices of *α*. Indeed, we can achieve the exact solutions of this problem for all choices of the fractional-order generalized Laguerre parameters *α*.

**Table 3:** The values *c*_0_, *c*_1_, *c*_2_, … and *c*_6_ for different values of *α* at $\nu =\frac{1}{2}$ for Example 2.

*α*	*c*_0_	*c*_1_	*c*_2_	*c*_3_	*c*_4_	*c*_5_	*c*_6_
0	718	-4316	10798	-14400	10800	-4320	720
1	5034	-15114	25200	-25200	15120	-5040	720
2	20150	-40310	50400	-40320	20160	-5760	720
3	60500	-90700	90700	-60500	25920	-6480	720

**Example 3** *We next consider the following problem*

$$\begin{array}{c} D^{\nu }u\left(x\right)+u\left(x\right)=g\left(x\right),\;\;u\left(0\right)=1,\;\;x\in \left[0,100\right], \end{array}$$ (74)

*where*

$$\begin{array}{c} g\left(x\right)=cos\left(\gamma x\right)+\frac{1}{\Gamma (-\nu )}\int_{0}^{x}{\left(x-t\right)}^{-\nu -1}u\left(t\right)dt \end{array}$$

*and the exact solution is given by* *u*(*x*) = *cos*(*γx*).

**Fig 1:**
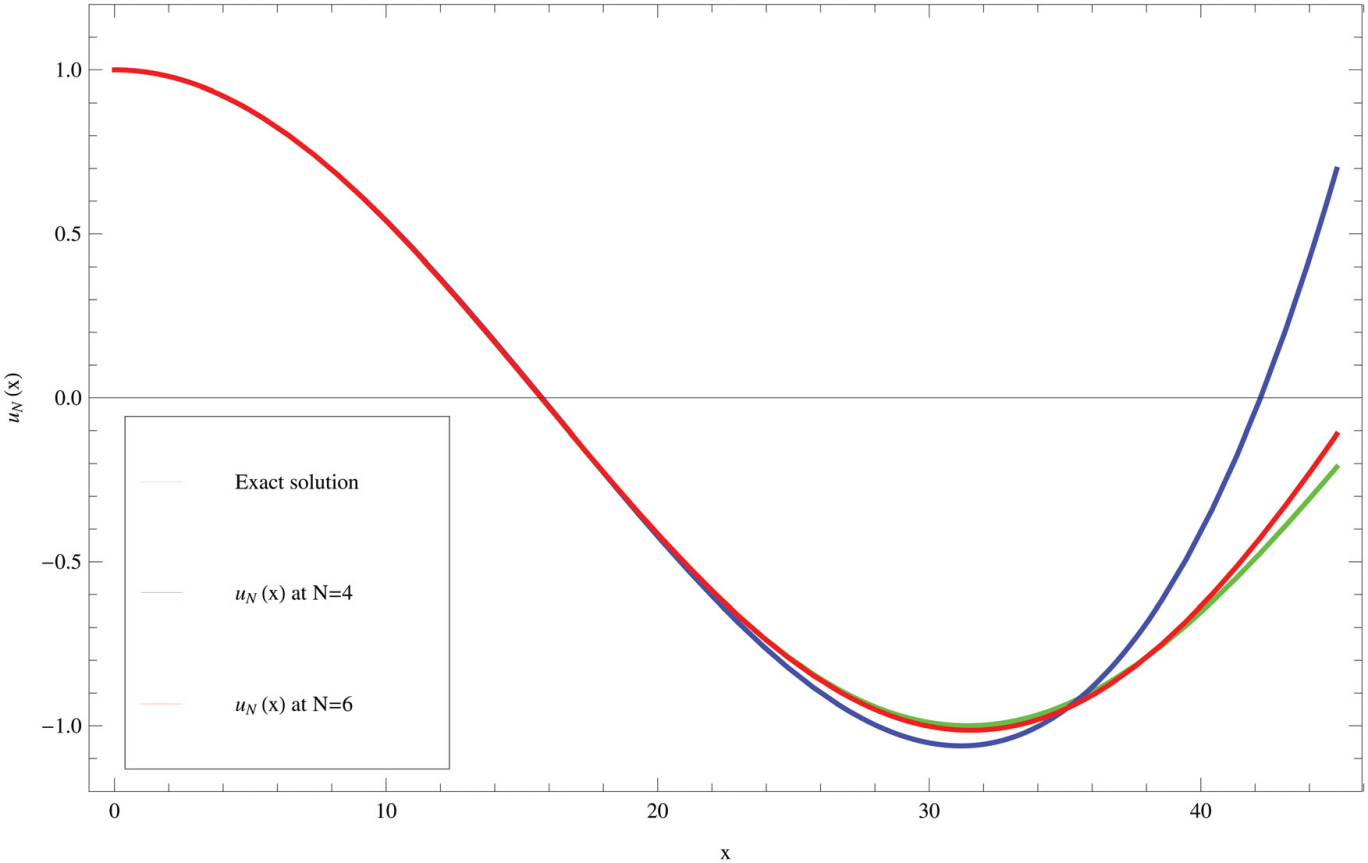
Comparing the exact solution and approximate solutions at *N* = 4, 6, where *α* = 0, $\lambda =\frac{3}{4}$ and *γ* = 0.1, for problem Eq (74).

The solution of this problem is obtained by applying the technique described in Section 6.2 based on the FGLOM of fractional integration. The maximum absolute error for $\gamma =0.01,\lambda =\frac{1}{2}$ and various choices of *N* and *α* are shown in Table 4. Moreover, the approximate solution obtained by the proposed method for $\alpha =0,\lambda =\frac{3}{4},\gamma =0.1$ and two choices of *N* is shown in Fig 1 to make it easier to compare with the analytic solution. From this figure, we see the coherence of the exact and approximate solutions.

**Table 4:** Maximum absolute error for *γ* = 0.01, $\lambda =\frac{1}{2}$ and different values of *N* and *α* in *x* ∈ [0, 100] for Example 3.

*N*	*α*	error	*α*	error	*α*	error	*α*	error
2		1.46.10^−2^		2.09.10^−2^		2.41.10^−2^		2.18.10^−2^
4	0	3.30.10^−3^	1	6.62.10^−3^	2	1.13.10^−2^	3	2.03.10^−2^
6		8.80.10^−4^		1.90.10^−3^		3.00.10^−3^		4.00.10^−3^
8		1.12.10^−16^		1.13.10^−16^		1.97.10^−16^		1.93.10^−16^

**Example 4** *Consider the following nonlinear initial value problem*

$$\begin{array}{c} D^{\nu }u\left(x\right)+2u^{2}\left(x\right)=\Gamma \left(\nu +2\right)x+2{\left(x^{\nu +1}\right)}^{2},\;\;0<\nu \leq 1, \end{array}$$

*whose exact solution is given by* *u*(*x*) = *x* ^*ν*+1^.

**Fig 2:**
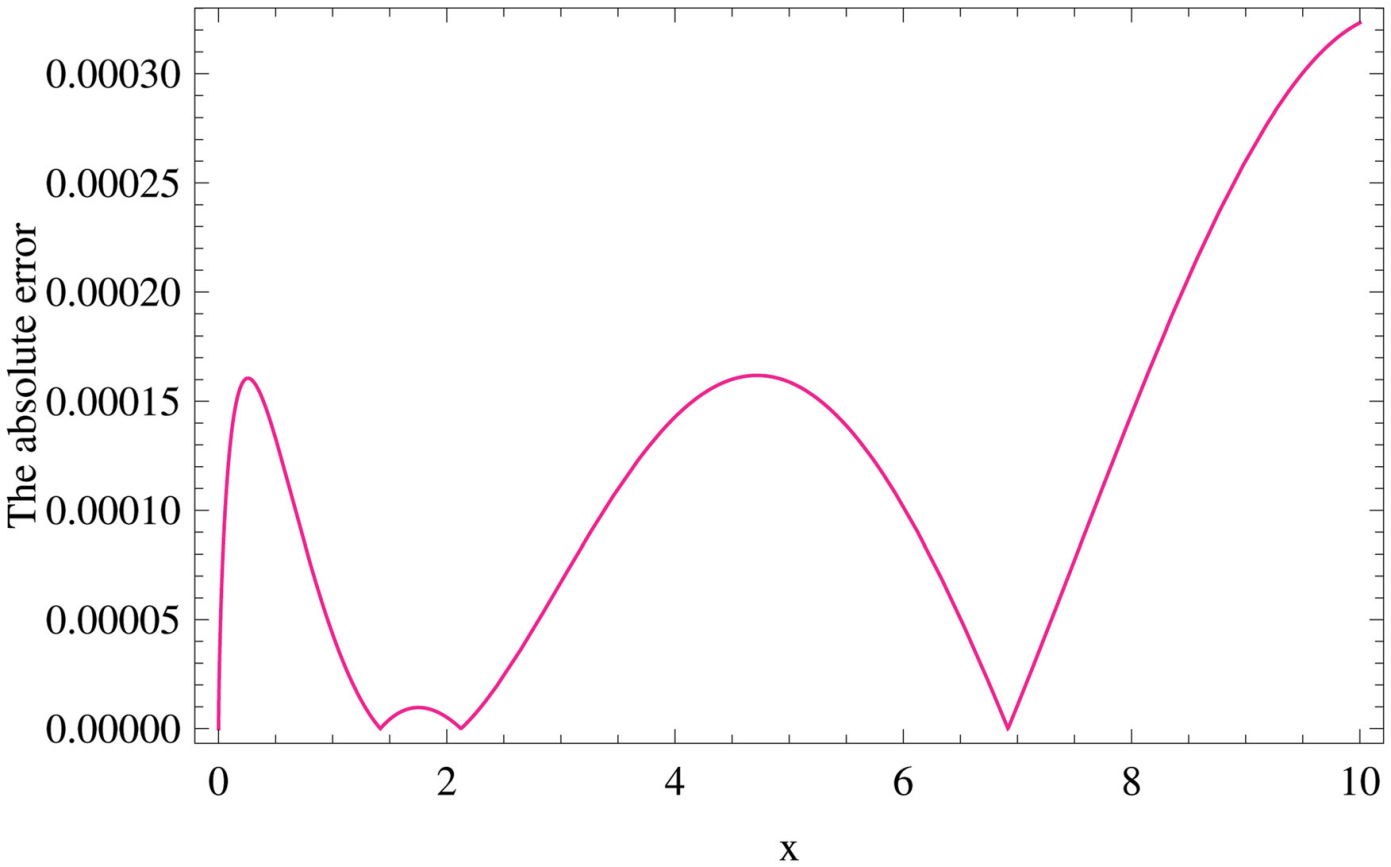
Graph of the absolute error function for *N* = 6, *α* = 0, $\lambda =\frac{3}{4}$ and *γ* = 0.1, for Example 4.

Table 5 shows the absolute error function of using spectral fractional-order generalized Laguerre collocation FGLC scheme in combination with FGLOM of fractional derivative with *ν*, *λ* and two choices of *α* at *N* = 16 in the interval [0, 40]. Fig 2 displays the absolute error function for *N* = 6, *α* = 0, $\lambda =\frac{3}{4}$ and *γ* = 0.1

**Table 5:** Maximum absolute error with various choices of *ν*, *λ* and *α* at *N* = 16 in *x* ∈ [0, 40], for Example 4.

*x*	*ν*	*λ*	*α* = 0	*α* = 2
1			1.19.10^−15^	1.80.10^−14^
10			1.04.10^−12^	1.10.10^−10^
20	0.5	0.5	9.84.10^−12^	1.88.10^−11^
30			2.80.10^−11^	3.88.10^−10^
40			1.97.10^−11^	1.07.10^−10^

**Example 5** *Consider the FDE*

$$\begin{array}{c} D^{2}u\left(x\right)+D^{\frac{3}{2}}u\left(x\right)+u\left(x\right)=x^{2}+2+\frac{\Gamma \left(3\right)}{\Gamma \left(\frac{3}{2}\right)}x^{\frac{1}{2}},\;\;u\left(0\right)=0,\;\;u^{\prime }\left(0\right)=0, \end{array}$$ (75)

*the exact solution is given by* *u*(*x*) = *x* ^2^.

We convert Eq (75) into a system of FDEs by changing variable *u*_1_(*x*) = *u*(*x*) obtaining:

$$\begin{array}{c} \begin{array}{c} D^{\frac{1}{2}}u_{1}\left(x\right)=u_{2}\left(x\right)\\ D^{\frac{1}{2}}u_{2}\left(x\right)=u_{3}\left(x\right)\\ D^{\frac{1}{2}}u_{3}\left(x\right)=u_{4}\left(x\right)\\ D^{\frac{1}{2}}u_{4}\left(x\right)=-u_{4}\left(x\right)-u_{1}\left(x\right)+x^{2}+2+\frac{\Gamma \left(3\right)}{\Gamma \left(1.5\right)}x^{\frac{1}{2}}, \end{array} \end{array}$$ (76)

with initial conditions

$$\begin{array}{c} u_{1}\left(0\right)=u\left(0\right),\;\;\;u_{2}\left(0\right)=0,\;\;\;u_{3}\left(0\right)=u^{\prime }\left(0\right),\;\;\;u_{4}\left(0\right)=0. \end{array}$$ (77)

The maximum absolute error for *y*(*x*) = *y*_1_(*x*) using FGLC method at *N* = 4 and various choices of *α* are shown in Table 6. It is clear that the approximate solutions are in complete agreement with the exact solutions.

**Table 6:** Maximum absolute error using FGLC method with various choices of *α* at *N* = 4 for Example 5.

*α*	E
$-\frac{1}{2}$	3.76.10^−14^
0	2.84.10^−14^
$\frac{1}{2}$	2.88.10^−14^
1	5.39.10^−13^
2	6.63.10^−14^
3	6.73.10^−14^

**Example 6** *Consider the initial value problem*

$$\begin{array}{c} D^{2}u\left(x\right)-D^{\left(\frac{3}{2}\right)}u\left(x\right)+\frac{6}{5}D^{\left(1\right)}u\left(x\right)+D^{\left(\frac{1}{2}\right)}u\left(x\right)+\frac{1}{5}u\left(x\right)=f\left(x\right),\;\;u\left(0\right)=0,\;\;u^{\prime }\left(0\right)=0, \end{array}$$ (78)

*with an exact solution* $u(x)=x^{\frac{5}{2}}+x^{2}$.

We convert Eq (78) into a system of FDEs by changing variable *u*_1_(*x*) = *u*(*x*) obtaining:

$$\begin{array}{c} \begin{array}{c} D^{\frac{1}{2}}u_{1}\left(x\right)=u_{2}\left(x\right)\\ D^{\frac{1}{2}}u_{2}\left(x\right)=u_{3}\left(x\right)\\ D^{\frac{1}{2}}u_{3}\left(x\right)=u_{4}\left(x\right)\\ D^{\frac{1}{2}}u_{4}\left(x\right)=u_{4}\left(x\right)-\frac{6}{5}u_{3}\left(x\right)-u_{2}\left(x\right)-\frac{1}{5}u_{1}\left(x\right)+f\left(x\right), \end{array} \end{array}$$ (79)

with initial conditions

$$\begin{array}{c} u_{1}\left(0\right)=u\left(0\right),\;\;\;u_{2}\left(0\right)=0,\;\;\;u_{3}\left(0\right)=u^{\prime }\left(0\right),\;\;\;u_{4}\left(0\right)=0. \end{array}$$ (80)

**Fig 3:**
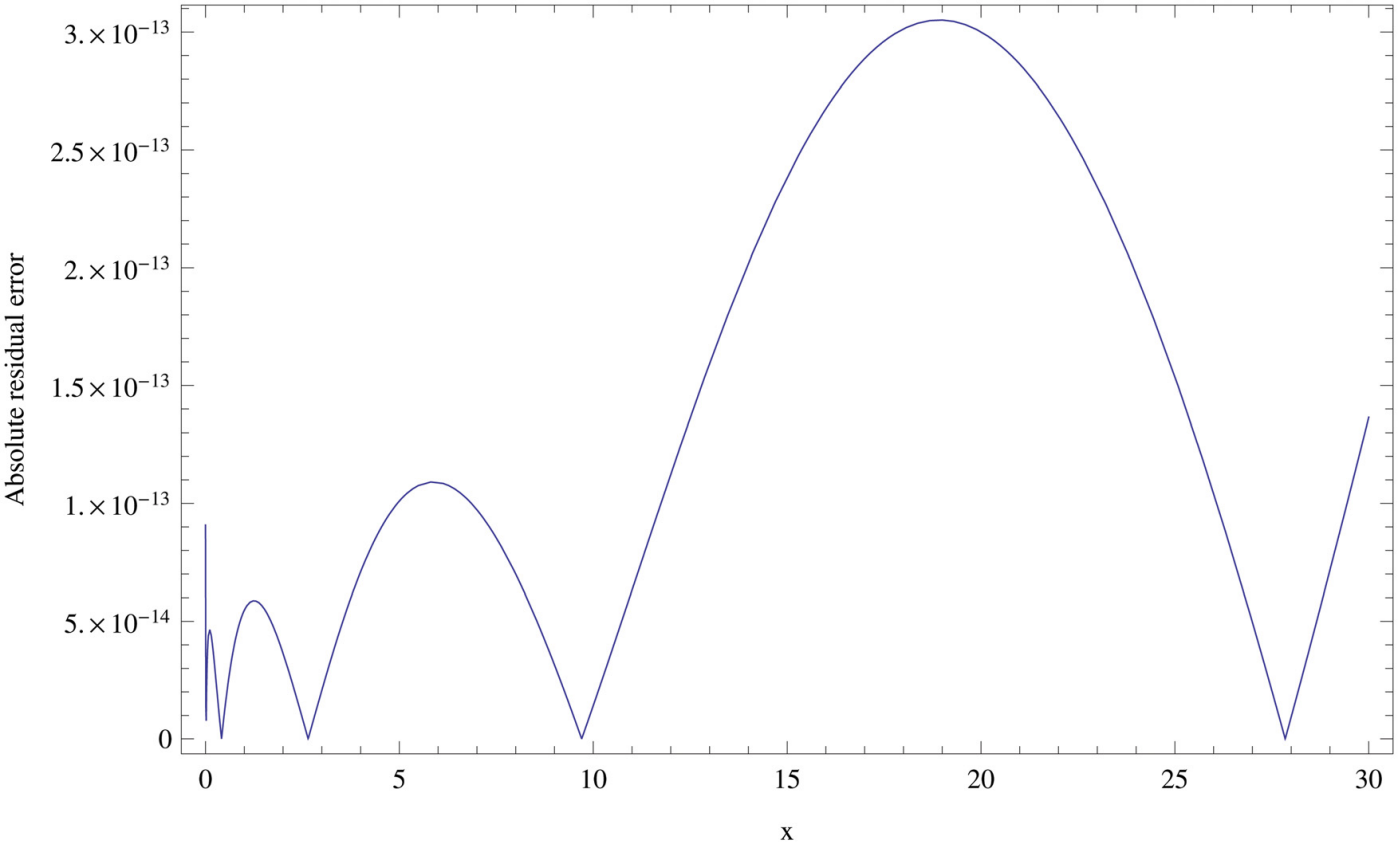
Graph of the absolute error function for *N* = 10, *α* = 0 and *ν* = *λ* = 0.5, for Example 6.

In Table 7, we list the results obtained by the fractional-order generalized Laguerre generalized collocation (FGLC) method with various choices of *α*, *N* = 10, and *ν* = *λ* = 0.5. The present method is compared with the shifted Chebyshev spectral tau (SCT) method given in [48]. As we see from Table 7, it is clear that the result obtained by the present method for each choice of the parameter *α* is superior to that obtained by SCT method. Fig 3 shows the absolute error function at *N* = 10, *α* = 0 and *ν* = *λ* = 0.5. The obtained results of this example show that the present method is very accurate by selecting a few number of fractional-order generalized Laguerre generalized functions.

**Table 7:** Absolute error using FGLC method with various choices of *α*, *N* = 10 and *ν* = *λ* = 0.5 for Example 6.

SCT (N = 64) [48]	FGLC method (N = 10)					
	$\alpha =-\frac{1}{2}$	*α* = 0	$\alpha =\frac{1}{2}$	*α* = 1	*α* = 2	*α* = 3
2.2.10^−8^	2.6.10^−10^	1.0.10^−13^	3.0.10^−12^	3.9.10^−12^	7.3.10^−12^	8.0.10^−12^

## Conclusion

We have defined new orthogonal functions namely FGLFs. The fractional operational matrices of Caputo fractional derivatives and Riemann-Liouville fractional integration were established for these functions. Two efficient spectral tau techniques were proposed based on these fractional operational matrices for solving linear FDEs of order *ν* (0 < *ν* < 1) on the half line.

In addition, we have developed the fractional-order generalized Laguerre pseudo-spectral approximation for solving the nonlinear initial value problem of fractional order *ν*. This technique was extended to solve systems of FDEs. The results of the proposed spectral schemes based on FGLFs were compared with other methods. Several numerical examples were implemented for FDEs and systems of FDEs including linear and nonlinear terms to demonstrate the high accuracy and the efficiency of the proposed techniques. The main idea and techniques developed in this work provide an efficient framework for the collocation method of various nonlinear FDEs on the half line. We also assert that the proposed technique can be extended to solve the one- and two-dimensional space/time fractional partial equations on the half line, (see [49–53]).

## References

[1] Zayernouri M, Karniadakis GE. Fractional Sturm-Liouville eigen-problems: Theory and numerical approximations, J. Comput. Phys. 47 (2013) 2108–2131.

[2] Ichise M, Nagayanagi Y, Kojima T. An analog simulation of non-integer order transfer functions for analysis of electrode processes, J. Electroanal. Chem Interfacial Electrochem 33 (2) (1971) 253–265. 10.1016/S0022-0728(71)80115-8

[3] Valerio D, Trujillo JJ, Rivero M, Machado JAT, Baleanu D. Fractional calculus: A survey of useful formulas, Eur. Phys. J. Special Topics 222 (2013) 1827–1846 10.1140/epjst/e2013-01967-y

[4] Bhrawy AH, Abdelkawy MA. A fully spectral collocation approximation for multi-dimensional fractional Schrodinger equations, Journal of Computational Physics, 294, (2015) 462–483 10.1016/j.jcp.2015.03.063

[5] Ye H, Liu F, Turner I, Anh V, Burrage K. Series expansion solutions for the multi-term time and space fractional partial differential equations in two and three dimensions, Eur. Phys. J., Special Topics, 222, (2013) 1901–1914. 10.1140/epjst/e2013-01972-2

[6] Sadati SJ, Ghaderi R, Ranjbar AN. Some fractional comparison results and stability theorem for fractional time delay systems, Romanian Reports in Physics, 65, (2013) 94–102.

[7] West BJ, Bologna M, Grigolini P. Physics of Fractal Operators, Springer-Verlag, New York, NY, 2003.

[8] Wang GW, Xu TZ. Symmetry properties and explicit solutions of the nonlinear time fractional KdV equation, Boundary Value Problems, 2013 (2013) 232 10.1186/1687-2770-2013-232

[9] Wang GW, Xu TZ. Lie symmetry analysis and explicit solutions of the time fractional fifth-order Kdv equation, Pols One, 2014 10.1371/journal.pone.0088336 PMC3921151 24523885

[10] Yi M, Huang J. Wavelet operational matrix method for solving fractional differential equations with variable coefficients, Applied Mathematics and Computation, 230 (2014) 383–394 10.1016/j.amc.2013.06.102

[11] Tohidi E, Nik HS. A Bessel collocation method for solving fractional optimal control problems, Applied Mathematical Modelling, 39 (2015) 455–465 10.1016/j.apm.2014.06.003

[12] Heydari MH, Hooshmandasl MR, Maalek Ghaini FM. An efficient computational method for solving fractional biharmonic equation, Computers and Mathematics with Applications, 68 (2014) 269–287 10.1016/j.camwa.2014.06.001

[13] Kumar D, Singh J. Sushila, Application of homotopy analysis transform method of fractional biological population model, Romanian Reports in Physics, 65, (2013) 63–75.

[14] Tenreiro Machado J. Numerical calculation of the left and right fractional derivatives, Journal of Computational Physics, (2014).

[15] Bhrawy AH, Zaky MA. A method based on the Jacobi tau approximation for solving multi-term time-space fractional partial differential equations, Journal of Comptuational Physics, 281 (2015), 876–895 10.1016/j.jcp.2014.10.060

[16] Zeng F, Liu F, Li C, Burrage K, Turner I, Anh V. Crank-Nicolson ADI spectral method for the two-dimensional Riesz space fractional nonlinear reaction-diffusion equation, SIAM Journal on Numerical Analysis, 52 (6) (2014) 2599–2622 10.1137/130934192

[17] Liu F, Meerschaert MM, McGough R, Zhuang P, Liu Q. Numerical methods for solving the multi-term time fractional wave equations, Fractional Calculus & Applied Analysis, 16 (2013) 9–25 23772179 10.2478/s13540-013-0002-2 PMC3679177

[18] Tong B, He Y, Wei L, Zhang X. A generalized fractional sub-equation method for fractional differential equations with variable coefficients, Physics Letters A, 376 (2012) 2588–2590 10.1016/j.physleta.2012.07.018

[19] Wang GW, Xu TZ. The modified fractional sub-equation method and its applications to nonlinear fractional partial differential equations, Romanian Journal of Physics, 66, (2014) 636–645.

[20] Bhrawy AH. An efficient Jacobi pseudospectral approximation for nonlinear complex generalized Zakharov system, Applied Mathematics and Computations, 247 (2014) 30–46 10.1016/j.amc.2014.08.062

[21] Chen F, Xu Q, Hesthaven JS. A multi-domain spectral method for time-fractional differential equations, Journal of Computational Physics, (2015) 10.1016/j.jcp.2015.03.033 25897178 PMC4400671

[22] Xiao-yong Z, Junlin L. Convergence analysis of Jacobi pseudo-spectral method for the Volterra delay integro-differential equations, Appl. Math. Info. Sci. 9 (2015) 135–145 10.12785/amis/090118

[23] Abdelkawy MA, Ahmed EA, Sanchez P. A method based on Legendre pseudo-spectral approximations for solving inverse problems of parabolic types equations, Math. Sci. Lett. 4 (2015) 81–90

[24] Tripathi MP, Baranwal VK, Pandey RK, Singh OP. A new numerical algorithm to solve fractional differential equations based on operational matrix of generalized hat functions, Commun. Nonlinear Sci. Numer. Simulat. 18 (2013) 1327–1340. 10.1016/j.cnsns.2012.10.014

[25] Doha EH, Bhrawy AH, Hafez RM. On shifted Jacobi spectral method for high-order multi-point boundary value problems, Commun. Nonlinear Sci. Numer. Simulat. 17 (2012) 3802–3810 10.1016/j.cnsns.2012.02.027

[26] Zayernouri M, Karniadakis GE. Fractional spectral collocation methods for linear and nonlinear variable order FPDEs, Journal of Computational Physics, (2015)

[27] Doha EH, Bhrawy AH, Hafez RM, Abdelkawy MA. A Chebyshev-Gauss-Radau scheme for nonlinear hyperbolic system of first order, Applied Mathematics and Information Science, 8 (2014) 535–544 10.12785/amis/080211

[28] Bhrawy AH, Zaky MA. Numerical simulation for two-dimensional variable-order fractional nonlinear cable equation, Nonlinear Dyn, 80 (1), (2015) 101–116. 10.1007/s11071-014-1854-7

[29] Zayernouri M, Karniadakis GE. Exponentially accurate spectral and spectral element methods for fractional ODEs, J. Comput. Phys. 257 (2014) 460–480. 10.1016/j.jcp.2013.09.039

[30] Ma X, Huang C. Spectral collocation method for linear fractional integro-differential equations, Appl. Math. Model. 38 (2014) 1434–1448. 10.1016/j.apm.2013.08.013

[31] Gottlieb D, Orszag A. Numerical Analysis of Spectral Methods: Theory and Applications, 1977.

[32] Mikhailenko BG. Spectral Laguerre method for the approximate solution of time dependent problems, Applied Mathematics Letters, 12 (1999) 105–110 10.1016/S0893-9659(99)00043-9

[33] Alici H, Taseli H. The Laguerre pseudospectral method for the radial Schrodinger equation, Applied Numerical Mathematics, 87 (2015) 87–99 10.1016/j.apnum.2014.09.001

[34] Xiao-Yong Z, Yan L. Generalized Laguerre pseudospectral method based Laguerre interpolation, Applied Mathematics and Computation, 219 (2012) 2545–2563 10.1016/j.amc.2012.08.090

[35] Gulsu M, Gurbuz B, Ozturk Y, Sezer M. Laguerre polynomial approach for solving linear delay difference equations, Applied Mathematics and Computation, 217 (2011) 6765–6776 10.1016/j.amc.2011.01.112

[36] Tatari M, Haghighi M. A generalized Laguerre-Legendre spectral collocation method for solving initial-boundary value problems. Applied Mathematical Modelling 38 (2014) 1351–1364 10.1016/j.apm.2013.08.008

[37] Baleanu D, Bhrawy AH, Taha TM. Two efficient generalized Laguerre spectral algorithms for fractional initial value problems, Abstract and Applied Analysis 2013 (2013). 10.1155/2013/546502

[38] Bhrawy AH, Alghamdi MM, Taha TM. A new modified generalized Laguerre operational matrix of fractional integration for solving fractional differential equations on the half line, Adv. Differ. Equ. 2012 (2012) 0:179. 10.1186/1687-1847-2012-179

[39] Szegö G. Orthogonal Polynomials, Am. Math. Soc. Colloq. Pub. 23 (1985).

[40] Funaro D. Polynomial Approximations of Differential Equations, Springer-Verlag, (1992).

[41] Bouzrara K, Garna T, Ragot J, Messaoud H. Decomposition of an ARX model on Laguerre orthonormal bases, ISA Transactions, 51 (2012) 848–860 10.1016/j.isatra.2012.06.005 22784371

[42] Khan S, Al-Gonah AA. Operational methods and Laguerre-Gould Hopper polynomials, Applied Mathematics and Computation, 218 (2012) 9930–9942 10.1016/j.amc.2012.03.080

[43] Alejandro L, Molano M. On asymptotic properties of Laguerre-Sobolev type orthogonal polynomials, Arab J Math Sci, 19 (2013) 173–186 10.1016/j.ajmsc.2013.01.001

[44] Conte D, Ixaru LGr, Paternoster B, Santomauroe G. Exponentially-fitted Gauss-Laguerre quadrature rule for integrals over an unbounded interval, Journal of Computational and Applied Mathematics, 255 (2014) 725–736 10.1016/j.cam.2013.06.040

[45] Ozarslan MA. On a singular integral equation including a set of multivariate polynomials suggested by Laguerre polynomials, Applied Mathematics and Computation, 229 (2014) 350–358 10.1016/j.amc.2013.12.050

[46] Drivera K, Muldoon ME. Common and interlacing zeros of families of Laguerre polynomials, Journal of Approximation Theory, (2014)

[47] Canuto C, Hussaini MY, Quarteroni A, Zang TA. Spectral Methods in Fluid Dynamics, Springer, New York, (1988).

[48] Bhrawy AH, Tharwat MM, Yildirim A. A new formula for fractional integrals of Chebyshev polynomials: Application for solving multi-term fractional differential equations, Appl. Math. Modell. 37 (2013) 4245–4252. 10.1016/j.apm.2012.08.022

[49] Yu Q, Liu F, Turner I, Burrage K. Numerical simulation of the fractional Bloch equations, Journal of Computational and Applied Mathematics 255 (2014) 635–651 10.1016/j.cam.2013.06.027

[50] Stokes PW, Philippa B, Read W, White RD. Efficient numerical solution of the time fractional diffusion equation by mapping from its Brownian counterpart, Journal of Computational Physics, 282 (2015) 334–344 10.1016/j.jcp.2014.11.023

[51] Zeng F. Second-order stable finite difference schemes for the time-fractional diffusion-wave equation, Journal of Scientific Computing, (2014) 10.1007/s10915-014-9966-2

[52] El-Ajou A, Arqub OA, Momani S. Approximate analytical solution of the nonlinear fractional KdV-Burgers equation: A new iterative algorithm, Journal of Computational Physics, (2014) 10.1016/j.jcp.2014.08.004

[53] Liu F, Zhuang P, Turner I, Anh V, Burrage K. A semi-alternating direction method for a 2-D fractional FitzHugh-Nagumo monodomain model on an approximate irregular domain, Journal of Computational Physics, (2015)

