# Correction: New Operational Matrices for Solving Fractional Differential Equations on the Half-Line

**DOI:** 10.1371/journal.pone.0138280

**Published:** 2015-09-14

**Authors:** Ali H. Bhrawy, Taha M. Taha, Ebrahim O. Alzahrani, Dumitru Baleanu, Abdulrahim A. Alzahrani

The affiliation for the fifth author is incorrect. Abdulrahim A. Alzahrani is not affiliated with #3, but with: Chemical and Materials Engineering Department, Faculty of Engineering, King Abdulaziz University, Jeddah, Saudi Arabia.
