# Correction: New Operational Matrices for Solving Fractional Differential Equations on the Half-Line

**DOI:** 10.1371/journal.pone.0142275

**Published:** 2015-10-30

**Authors:** Ali H. Bhrawy, Taha M. Taha, Ebraheem O. Alzahrani, Dumitru Baleanu, Abdulrahim A. Alzahrani

The third author’s name is misspelled in the original article and in the correction published on September 14, 2015. The correct name is: Ebraheem O. Alzahrani.
